# Bichloride of Mercury in Dental Practice

**Published:** 1888-08

**Authors:** Louis Ottofy

**Affiliations:** Chicago, Ill.


ARTICLE III.

### BICHLORIDE OF MERCURY IN DENTAL PRACTICE.

BY LOUIS OTTOFY, D. D. S., CHICAGO, ILL.

(Read before the Southern Illinois Dental Society. April, 1888.)

The dividing line which is supposed to have existed between the animal and vegetable kingdoms is being rapidly obliterated, indeed it is now almost beyond question that there is not sufficient difference to justify any division into separate kingdoms. As in our observations we descend to the lower class of animals and plants, some of each are reached which have so few and slightly distinguishing features that they may be classified with either animals or plants, without in any marked degree violating the laws governing either species. To this vast class multitudes of minute living beings belong. The air and water is filled and peopled with them. In the lowest species of plants we find the various disease germs and spores, which have been erroneously designated as animalcules, but are now properly known to be plants. The closer knowledge we now have of the life and habits of these germs has entirely revolutionized medical science and practice. Diseases whose origin has thus been discovered, and diseases whose management has been beyond human control, are now comparatively well understood. Undoubtedly the introduction of these germs into the system under various conditions and at various times lead to certain specific results. Nor is this less true of that portion of the human body more directly under the care of the dentist. It has been ascertained that the destruction of these germs and their exclusion from diseased parts, or indeed from the entire organism, is followed by favorable changes, and that usually a return to physiological conditions is the result. There are a number of chemicals and drugs which are said to be capable of bringing about these favorable results, and foremost among them is classed the bichloride of mercury.

The uses of this agent in dental practice are almost endless; it can be safely resorted to in all operations in which the result depends on the destruction of spores or microbes, which are now generally accepted to be instrumental in creating or maintaining diseased conditions. It can be used in solution of from one part in two hundred of water to one part in two thousand. A stronger solution than the former may not be safe, and weaker solutions than the latter are not supposed to be effective for the sterilization essential in dental surgery. The best method of having a stock solution on hand is to take 100 grains mercuric bichloride and add it to 1,000 parts of water at one time. This quantity requires the addition of alcohol, as the bichloride is not soluble in water to the extent of 100 in 1,000. This may be kept in a cool and dark place, and from it three solutions should be made, namely:

One of one part in 200
" " " 500 and
" " " 1,000, in quantities regulated by the amount of each as may be required. The 100, 500 and 1,000 solutions are used only in small quantities, hence an ounce vial of each in the operating case is all that is necessary. To prepare them take from the pint bottle

Ten parts to 190 of water: makes one in 200
" " 490 " " " 500 and
" " 990 " " " 1,000.

A tumbler, bottle or small pitcher full of the latter should be made each day for the washstand to be used as it is, or it may be diluted with one-half of water, making a 1 in 2,000 solution to be used for purposes mentioned hereafter.

The dose of bichloride of mercury is from 1-20 to ¼ of a grain. In the solution prepared the following will be the proportions:

| Solution. | Per Cent. of Bichl. of Mer. | In 100 drops. | In 50 drops. | In 10 drops. |
|---|---|---|---|---|
| One in 200 | ½ | ½ grains | ¼ grains | 1-20 grains |
| One in 500 | 1-5 | 1-5 grains | 1-10 grains | 1-50 grains |
| One in 1,000 | 1-10 | 1-10 grains | 1-20 grains | 1-100 grains |
| One in 2,000 | 1-20 | 1-20 grains | 1-40 grains | 1-1000 grains |

In opening into a cavity for any purpose whatever, and whether after or before the application of the rubber dam, the cavity having been dried should be flooded with 1 in 1,000 solution. This solution should be used for sterilizing exposed pulps and disinfecting cavities when they extend near to the pulp. It is the solution *par excellence* to be used in implantation; in it all instruments used in this operation should be dipped and the hands, towels, syringes and anything else which may come in contact with the tooth or mouth should be moistened with it, for success in implantation depends mainly on perfect sterilization and healing of the parts by first intention. The foregoing table demonstrates that the minimum dose of the drug is contained in fifty drops of the solution (1 in 1,000), hence no fear need be entertained in regard to the poisonous action of the drug, as that quantity would have to be swallowed or otherwise taken into the system in order to administer the minimum dose, and 150 drops to get the effect of the maximum dose.

The 1 in 200 and the 1 in 500 solutions are to be used carefully; the former contains the minimum dose of the salt in 10 drops and the latter in 50 drops. These solutions are used principally in root-canals, and the quantity and strength to be used should be governed by the size of the dental foramen, as is generally indicated by the instrument used or by the age of the patient. In cases where so-called immediate root filling is practiced, the use of bichloride of mercury is essential. Its rapid and certain action in destroying disease germs and its preservative quality are brought into prominence. In teeth where the dental foramen is large, the 1-5 per cent. solution (1 in 500) should be used cautiously, while the 1-10 per cent. solution (1 in 1,000) may be used with impunity. The ½ per cent. solution (1 in 200) is used in root-canals whose foramina are small. But the principal use I have found for this solution is in painting those small irritable patches, which cause so much annoyance to those afflicted with them. These mucous plaques seem to have a certain course to run. I do not know to what causes they are due, or how they can be cured. They begin as a small irritating red spot, almost anywhere on the mucous membrane of the mouth or tongue, become gradually larger, generally about the one-twelfth of an inch in diameter, their surface is white or grey, and when in contact with the teeth, or when situated in folds of the mucous membrane, are very tender and painful; on the third or fourth day they are generally unbearable, and by the seventh or eighth have entirely disappeared. By touching these spots three or four times a day with the 1 in 200 solution, their progress is generally checked, though in some cases merely improved, but in either case they are less painful and disagreeable.

The 1-2 per cent. (1 in 2,000) solution is used for washing the hands, spittoon and instruments; the latter are dipped in it and rapidly wiped and rubbed clean, and when thus treated do not corrode. Of the forceps generally only the beaks need thus be treated. In this strength it may also be used as a mouth-wash, half of the solution and half of listerine, adding a few drops of the extract of white rose, jockey club, Mary Stuart or any other sold in the drug stores. These extracts and the listerine combined, to some extent at least, disguise the disagreeable taste and astringent action of the salt. These solutions have to be used thus without a definite knowledge of their exact action until it is definitely ascertained in what degree they are effective, and for what length of time they must be kept in contact with the parts to be acted on. For the present, at least, it is certain that no known substance has the properties of destroying parasites in so marked a degree as the bichloride of mercury. Its poisonous effect and its tendency to corrode instruments is the principal objection to its use; both of these can, in a measure, be overcome, and, with the exercise of good judgment, the use of bichloride of mercury will prove to be of much benefit to those who have not used it, or who have resorted to its use to a limited extent only. —*Dental Review*.

